# NK cell recruitment limits tissue damage during an enteric helminth infection

**DOI:** 10.1038/s41385-019-0231-8

**Published:** 2019-11-27

**Authors:** Maria E. Gentile, Yue Li, Amicha Robertson, Kathleen Shah, Ghislaine Fontes, Eva Kaufmann, Barbara Polese, Nargis Khan, Marc Parisien, Hans M. Munter, Judith N. Mandl, Luda Diatchenko, Maziar Divangahi, Irah L. King

**Affiliations:** 1 Meakins-Christie Laboratories, Department of Medicine, McGill University Health Centre, Montreal, QC H4A 3J1 Canada; 2 Department of Microbiology and Immunology, McGill University, Montreal, QC H3A 2B4 Canada; 3 McGill International TB Centre, McGill University Health Centre, Montreal, QC H4A 3J1 Canada; 4 Alan Edwards Centre for Research on Pain, Department of Anesthesia, McGill University, Montreal, QC H3A 0G1 Canada; 5 Department of Human Genetics, McGill University Innovation Centre, Montreal, QC H3A 0G1 Canada; 6 Department of Physiology, Complex Traits Group, McGill University, Montreal, QC H3G 0B1 Canada; 7 Present Address: NYU Medical School, 550 First Avenue, New York, NY 10016 USA; 8 Present Address: Francis Crick Institute, 1 Midland Road, London, NW1 1AT England

## Abstract

Parasitic helminths cause significant damage as they migrate through host tissues to complete their life cycle. While chronic helminth infections are characterized by a well-described Type 2 immune response, the early, tissue-invasive stages are not well understood. Here we investigate the immune pathways activated during the early stages of Heligmosomoides polygyrus bakeri (Hpb), a natural parasitic roundworm of mice. In contrast to the Type 2 immune response present at later stages of infection, a robust Type 1 immune signature including IFNg production was dominant at the time of parasite invasion and granuloma formation. This early response was associated with an accumulation of activated Natural Killer (NK) cells, with no increase of other innate lymphoid cell populations. Parabiosis and confocal microscopy studies indicated that NK cells were recruited from circulation to the small intestine, where they surrounded parasitic larvae. NK cell recruitment required IFNγ receptor signaling, but was independent of CXCR3 expression. The depletion of tissue-infiltrating NK cells altered neither worm burden nor parasite fitness, but increased vascular injury, suggesting a role for NK cells in mediating tissue protection. Together, these data identify an unexpected role for NK cells in promoting disease tolerance during the invasive stage of an enteric helminth infection.

## Introduction

Parasitic helminths are a neglected tropical disease, infecting > 25% of the world’s population. These macroparasites cause significant tissue damage as they migrate through host tissues to complete their life cycle and, as a result, may lead to morbidities such as intestinal bleeding and tissue fibrosis.^1^ Despite the negative impact on tissue physiology, many helminth species have co-evolved with their host species resulting in a symbiotic relationship. As such, the human parasites *Ascaris lumbricoides* or *Trichuris trichiura* and rodent parasites *Heligmosomoides polygyrus bakeri* (*Hpb*) or *Trichuris muris*, cause minimal clinical signs of illness, except in the case of high parasite load.^2^ This host–parasite relationship indicates that mammals have developed effective strategies to maintain tissue fitness during infection.

The chronic stages of helminth infection most commonly result in a robust Type 2, tissue reparative immune response. This response is characterized by high levels of Th2 cytokines such as interleukin (IL)-4, IL-5, and IL-13, tissue eosinophilia, the generation of alternatively activated macrophages and goblet cell secretion of mucus that collectively promote restoration of barrier integrity, repair of mucosal tissue, and worm expulsion.^3^ Conversely, Type 1 responses are generally characterized by production of interferon (IFN)γ and reactive oxygen species that limit epithelial cell turnover, increase inflammatory cell death pathways and prevent worm clearance.^4^ This dichotomy is well illustrated in the setting of *T. muris* infection, where resistant mouse strains produce a robust Type 2 immune response leading to parasite clearance, whereas susceptible strains mount a Type 1-dominated response resulting in chronic infection.^4,5^ An early Type 1 response has also been observed following infection with the helminth *Schistosoma mansoni*, but it is later subdued by an egg antigen-driven Type 2 immune response that promotes a protective granuloma response in the liver and intestinal tissues.^6^ Notably, unchecked Type 1 or Type 2 immunity during *T. muris* or *S. mansoni* infection can lead to increased mortality.^7,8^ Thus, a more nuanced balance between Type 1 and 2 immunity may be needed to maximize host defense during helminth infections.

*Hpb* is a natural parasitic nematode of mice that follows a reproducible kinetic of larval invasion into the proximal small intestinal submucosa to complete its life cycle. Upon maturation, adult worms emerge from the wall of the duodenum and intertwine themselves in the intestinal villi as egg-laying adults.^9^ Although previous studies described an exclusive Type 2 immune-dominated response to this parasite, a recent study described a role for IFNγ in promoting epithelial stem cell regeneration in the vicinity of the *Hpb* granuloma.^10^ These results led us to hypothesize that induction of an early Type 1 immune response limits tissue damage during the invasive stages of *Hpb* infection.

To test this hypothesis, we performed a kinetic analysis of the innate immune response during *Hpb* infection. We identified an IFNγ-dependent Type 1 immune gene signature as early as 2 days post infection (dpi) that was associated with a previously unidentified accumulation of IL-7Rα(CD127)^−^Eomesodermin (Eomes)^+^ natural killer (NK) cells at the site of infection. Parabiosis and immunophenotyping experiments determined that NK cell accumulation resulted from the recruitment of a circulating CD49a^−^CD49b^+^ population. Notably, IFNγ signals were crucial for NK cell recruitment, but this occurred independently of CXCR3 expression. Depletion of circulating NK cells did not impact adult worm burden or parasite fitness, but led to an increase in intestinal bleeding as well as activated platelet gene expression. Collectively, these data identify Type 1 immunity and *bona fide* NK cells as part of an acute damage control response to an enteric helminth infection that could be harnessed to minimize infection-induced tissue damage in the intestine.

## Results

### *Hpb* infection induces a rapid accumulation of *bona fide* NK cells in the small intestine

**Fig. 1:**
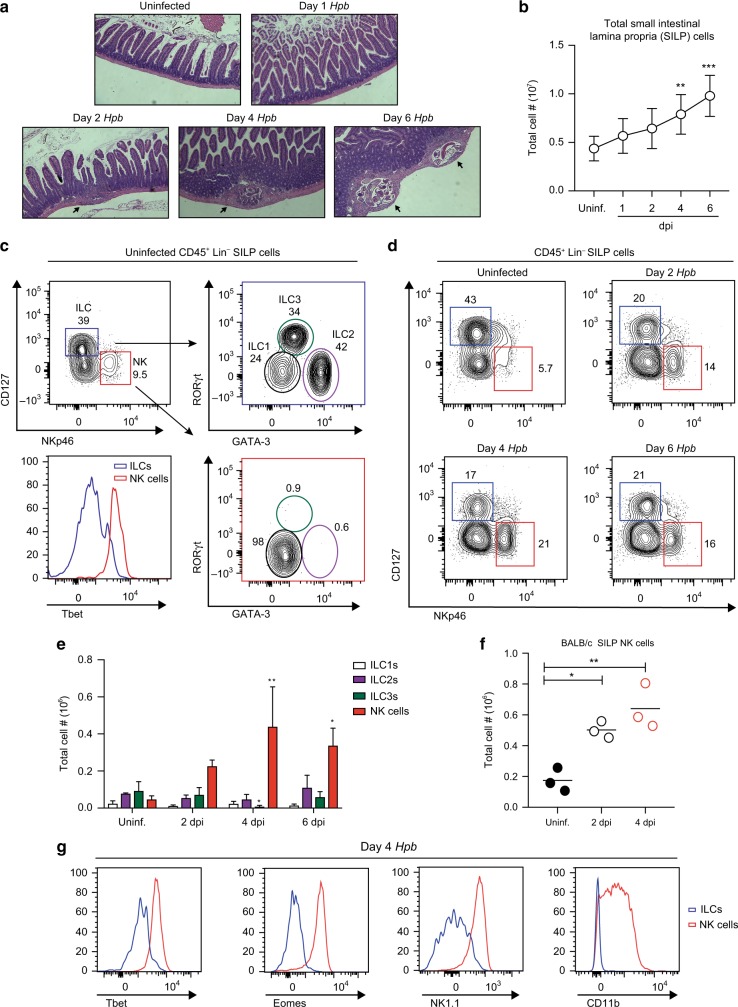
NK cells increase in the SILP during the early stages of *Hpb* infection. **a** Representative H&E staining of paraffin-embedded duodenal tissue of uninfected (Uninf.), 1, 2, 4, and 6 dpi of wild type (WT) mice with *Hpb* (× 5 magnification). Arrows point to detectable granulomas. **b** Total small intestinal lamina propria (SILP) cell number over the course of infection. **c** Representative contour plots of ILCs defined as CD45^+^CD3^−^B220^−^(Lin^−^)CD127^+^NKp46^−^ cells within the blue gate and NK cells defined as CD45^+^Lin^−^CD127^−^NKp46^+^ within the red gate, in the SILP of an uninfected WT mouse. Further gating of CD127^+^NKp46^−^ cells defined ILC1 (GATA3^−^RORγt^−^, in black), ILC2 (GATA3^+^RORγt^−^, in purple) and ILC3 (GATA3^int^RORγt^+^, in green) populations. Representative histograms of Tbet expression in ILCs and NK cells. **d** Representative contour plots of SILP ILCs and NK cells in uninfected, 2, 4, and 6 dpi of mice with *Hpb*. **e** Total cell numbers of ILC1s, ILC2s, ILC3s, and NK cells isolated from the SILP of uninfected mice or at 2, 4, and 6 dpi with *Hpb* (*n* = 4). **f** Total number of CD45^+^Lin^−^CD127^−^NKp46^+^ NK cells in the SILP of uninfected and *Hpb-*infected BALB/c mice. Each dot represents an individual mouse. **g** Representative histograms showing Tbet, Eomes, NK1.1, and CD11b expression by SILP ILCs or NK cells at 4 dpi with *Hpb*. **b**, **e** Data shown are pooled from three independent experiments. **b**, **f** Data were analyzed by one-way ANOVA with Dunnett’s post test for multiple comparisons, using uninfected as the control group. **e** Data were analyzed by one-way ANOVA with Dunnet’s post test for multiple comparisons for each cell type, using uninfected as the control group (*p < 0.05, **p < 0.01, ***p < 0.001). Error bars, SD.

*Hpb* infection follows a well-defined life cycle within the host. Upon entry into the proximal small intestine, specifically the duodenum, infectious larvae cross the epithelial barrier and embed within the submucosa within 24–48 h.^9^ This early tissue-invasive stage leads to an accumulation of immune cells within the small intestinal lamina propria (SILP), the formation of granulomas, and the maturation of larvae into adult worms prior to their re-emergence into the intestinal lumen beginning at day 6 post infection (Fig. 1a, b). To understand which immune cell types respond at early stages of infection, we first characterized the tissue-resident innate lymphoid cell (ILC) population in the SILP.^11^ In uninfected wild type (WT) C57BL/6 mice, ILC1s (Lin^−^NKp46^−^CD127^+^Tbet^+^), ILC2s (Lin^−^NKp46^−^CD127^+^GATA3^+^), and ILC3s (Lin^−^NKp46^−^CD127^+^RORγt^+^) were prominent while a small population of NK cells (Lin^−^CD127^−^NKp46^+^) was also present (Fig. 1c). However, upon *Hpb* infection, a striking accumulation of NK cells occurred as early as 2 dpi, which continued to increase through day 4 and plateaued by day 6 post-infection (Fig. 1d–e). Notably, no other ILC populations accumulated at these time points and, in fact, we observed a small, but significant decrease in ILC3s at 4 dpi relative to uninfected mice (Fig. 1d–e). Going forward, we focused on the first 4 days of infection as this time point represents the peak tissue-dwelling stage of *Hpb* and also preceded a detectable number of IL-4-producing SILP Th2 cells, representing onset of the adaptive immune response (Fig. S1).^12^ To test whether *Hpb*-induced NK cell accumulation was dependent on the genetic background of the mice, we also infected BALB/c mice. Similarly, NK cells accumulated in the SILP at 2 and 4 dpi with *Hpb*, demonstrating that this response was not limited to mice on the C57BL/6 background (Fig. 1f).

To confirm that the CD127^−^NKp46^+^ cells during *Hpb* infection of WT mice were in fact *bona fide* NK cells, we examined the expression of Tbet and Eomes, transcription factors that are required for NK cell development and maturation, respectively.^13^ Compared with CD127^+^ ILCs, CD127^−^NKp46^+^ NK cells in the SILP at 4 dpi expressed more Tbet and Eomes (Fig. 1g). In addition, this cell population also expressed the maturation marker CD11b and the activation receptor NK1.1 (Fig. 1g).^14^ Therefore, these data demonstrate that *bona fide* mature NK cells accumulate in the SILP during *Hpb* infection.

### Activated intestinal NK cells localize to the *Hpb* granuloma and accumulate independent of RORγt-expressing ILCs

**Fig. 2:**
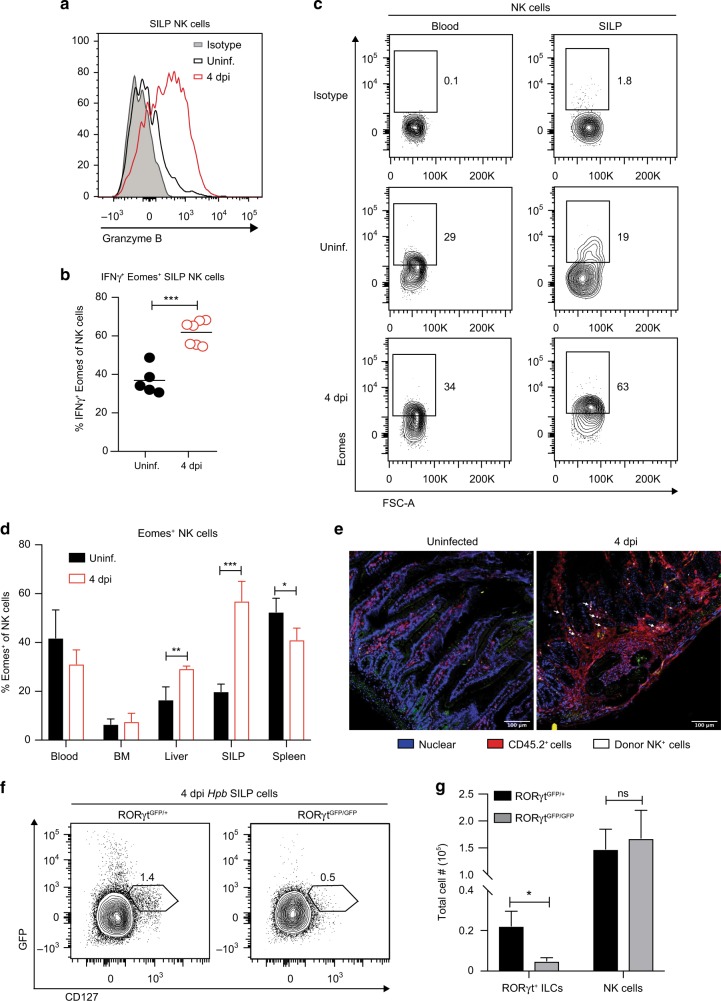
Activated NK cells localize to the *Hpb* granuloma and are not derived from a RORγt-expressing precursor. **a** Representative histogram showing Granzyme B expression in SILP Lin^−^CD11b^lo/int^CD127^−^NK1.1^+^ NK cells from uninfected mice and 4 dpi with *Hpb*. Isotype shown in shaded histogram. **b** Frequencies of CD127^−^NK1.1^+^Eomes^+^IFNγ^+^ NK cells in the SILP of uninfected and *Hpb-*infected mice after in vitro restimulation with PMA and Ionomycin in the presence of GolgiStop. Each dot represents an individual mouse. **c** Representative contour plots of CD127^−^NK1.1^+^ Eomes^+^ NK cells in the blood and SILP of uninfected mice and 4 dpi; top plot shows Eomes isotype control labeling. **d** Frequencies of CD127^−^NK1.1^+^Eomes^+^ NK cells in the blood, bone marrow (BM), liver, SILP, and spleen of uninfected mice and 4 dpi (*n* = 3-4). **e** Purified CD45.1^+^CFSE^+^-labeled NK cells were i.v. transferred into CD45.2^+^ recipient mice prior to *Hpb* infection. Representative duodenal sections of uninfected and *Hpb*-infected mice are shown. Nuclear stain in blue, CD45.2^+^ cells in red, and donor (CD45.1^+^ CFSE^+^) NK cells in white, scale bar = 100 μm. Arrows point to labeled NK cells. **f** Representative contour plots of CD45^+^Lin^−^CD127^+^GFP^+^ cells, and **g** total numbers of CD127^+^GFP^+^ ILCs and CD127^−^NKP46^+^ NK cells in the SILP of uninfected and 4 day *Hpb-*infected RORγt ^GFP/+^ and RORγt^GFP/GFP^ littermate controls mice (*n* = 3). **b** Data shown are pooled from two independent experiments. **b, d**, **g** Data were analyzed using an unpaired parametric *t* test comparing each infected group to its respective uninfected group (*p < 0.05, **p < 0.01, ***p < 0.001). Error bars, SD. NS, not significant.

NK cells are classically known for their role in viral infections, during which they produce Type 1 cytokines such as IFNγ and exert cytotoxic function to kill infected cells.^15^ We therefore asked whether NK cells present in the SILP during *Hpb* infection had the potential to exert classic NK cell effector functions. We found that at 4 dpi, SILP NK cells expressed the cytolytic molecule Granzyme B and had the capacity to produce IFNγ upon *ex vivo* restimulation, suggesting this population has the potential to retain its classical effector functions in the context of an anti-parasitic immune response (Fig. 2a, b).

We next determined whether the accumulation of mature NK cells during *Hpb* infection was specific to the SILP or whether this reflected systemic changes to the NK cell compartment. We therefore assessed the blood, bone marrow (BM), liver, SILP and spleen for changes in the NK cell population at 4 dpi with *Hpb*. As described above, we observed a significant increase in the frequency of Eomes^+^ NK cells in the SILP relative to uninfected mice (Fig. 2c, d). By contrast, no change in Eomes^+^ NK cells was detected in the blood or BM upon infection (Fig. 2c, d). Interestingly, small, but significant differences in the frequency of Eomes^+^ NK cells were observed in the spleen and liver following *Hpb* infection, suggesting a potential tissue redistribution. Although we did observe a significant increase in Eomes^+^ NK cells in the liver, this result was variable across experiments. We next wanted to localize the *Hpb*-induced NK cells within the SILP. To this end, we purified splenic NK cells from CD45.1^+^ mice, labeled them with CFSE and transferred them into CD45.2^+^-recipient mice 1 day prior to infection (Fig. S2A). At 4 dpi, we detected donor (CD45.1^+^CFSE^+^) NK cells in the blood and infected SILP (Fig. S2B). Using confocal microscopy, we found CFSE^+^ NK cells were localized within the vicinity of the granuloma (Fig. 2e). Therefore, a mature, activated NK cell population rapidly accumulates at the site of *Hpb* infection.

A previous study has shown that, during intestinal inflammation, NK-like cells can emerge from RORγt^+^ ILCs that have downregulated the transcription factor RORγt and increased expression of IFNγ.^16^ To assess if the NK cell population we observed was derived from a RORγt^+^ ILC subset, we infected mice in which *Gfp* is knocked-in to the endogenous *Rorc* locus (RORγt^GFP/GFP^), rendering mice homozygous for the transgene deficient in functional RORγt expression. *Hpb*-infected RORγt^GFP/+^ heterozygous littermates were used as controls. As expected, we noted a clear loss of RORγt^+^ ILCs in the SILP of RORγt^GFP/GFP^ mice compared with RORγt^GFP/+^ mice (Fig. 2f, g). In contrast, there was no change in the number of SILP NK cells in RORγt^GFP/GFP^ mice relative to littermate controls at 4 dpi (Fig. 2g). Together, these data demonstrate that NK cells selectively accumulate in the SILP during *Hpb* infection independent of a RORγt-expressing precursor cell.

### *Hpb* infection recruits circulating NK cells to the small intestine

**Fig. 3:**
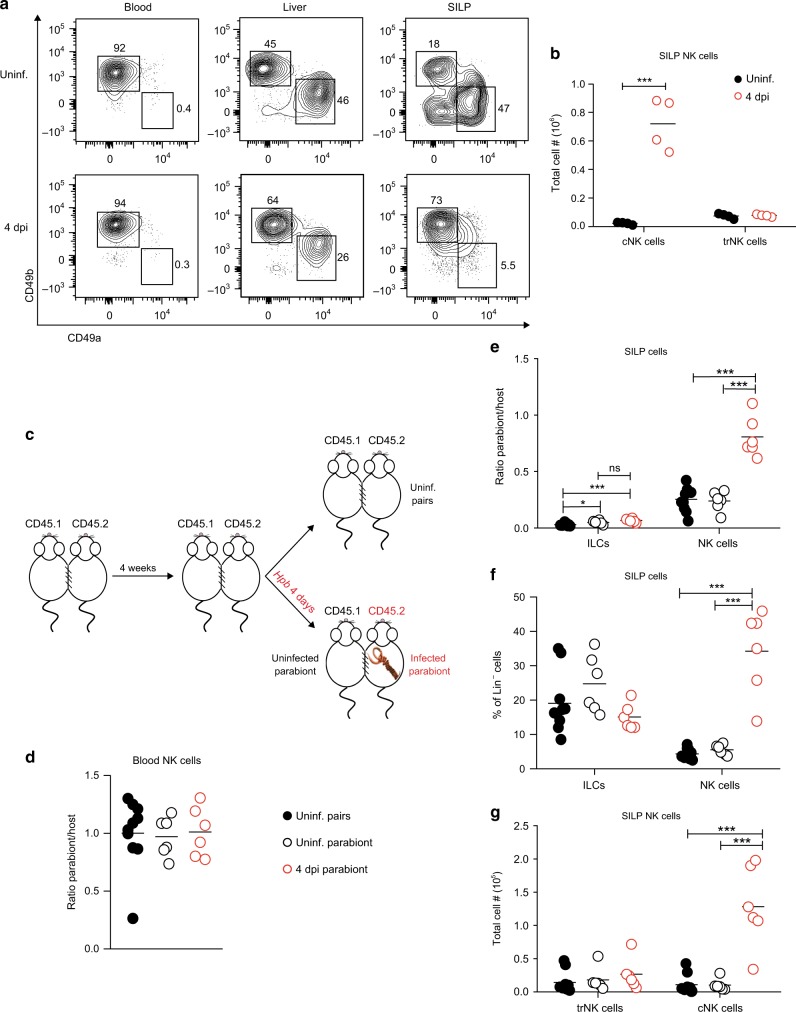
*Hpb* infection recruits circulating NK cells to the small intestine. **a** Representative contour plots of cNK cells (CD127^−^NK1.1^+^CD49a^−^CD49b^+^) and trNK cells (CD127^−^NK1.1^+^CD49a^+^CD49b^−^) in the blood, liver, and SILP of uninfected mice and 4 dpi with *Hpb*. **b** Total cell numbers of cNK and trNK cells in the SILP of uninfected mice and 4 dpi with *Hpb*. **c** Experimental design outlining the parabiosis experiments. **d** Ratio of Parabiont (CD45.1)/Host (CD45.2) Lin^−^ NK cells in the blood of uninfected and 4 dpi *Hpb* parabiotic mice. In uninfected pairs, data from CD45.1^+^ and CD45.2^+^ parabionts were pooled. In the infected pairs, the “host” represents the CD45.2^+^ mouse. **e** Ratio of Parabiont/Host ILCs (CD127^+^NK1.1^−^) and NK cells (CD127^−^NK1.1^+^) and **f** frequencies of ILCs and NK in the SILP of uninfected and infected parabiotic mice. **g** Total numbers of trNK and cNK cells in the SILP of uninfected and infected parabiotic mice. **d**–**g** Data shown are pooled from two independent experiments. **b**) Data was analyzed using an unpaired parametric *t* test for each cell type comparing with its respective uninfected group. **d**–**g** Data were analyzed by one-way ANOVA with Tukey’s post test for multiple comparisons (*p < 0.05, ***p < 0.001). Each dot represents an individual mouse. NS, not significant.

To further investigate the origin of *Hpb*-induced Eomes^+^ NK cells, we sought to determine whether they were derived from a tissue-resident or circulating population. Sojka et al.^17^ demonstrated that murine tissue-resident NK (trNK) cells and circulating NK (cNK) cells can be distinguished based on the cell surface expression of the integrin subunits CD49a and CD49b, respectively. Although they identified these subsets in multiple lymphoid and non-lymphoid tissues, the intestine was not examined.^17^ Similar to this study, CD49a and CD49b could be used to distinguish different NK cell subsets in the SILP at steady state (Fig. 3a). Consistent with previous reports, blood NK cells were exclusively CD49a^−^CD49b^+^ whereas the liver harbored both CD49a^+^CD49b^−^ trNK and CD49a^−^CD49b^+^ cNK cell subsets (Fig. 3a). Similar to the liver, the SILP of uninfected mice contained both cNK and trNK cell populations based on integrin subunit expression (Fig. 3a). However, following *Hpb* infection, there was a significant increase in the frequency and total number of cNK cells in the SILP, with no change in SILP trNK cell numbers compared with uninfected mice (Fig. 3a, b).

In order to confirm the origin of SILP NK cells, we generated congenically mismatched parabiotic mice. In this procedure, CD45.1^+^ and CD45.2^+^ mice were surgically joined to allow the sharing of the circulatory system (Fig. 3c). Four weeks after surgical joining, the CD45.2^+^ parabiont was infected with *Hpb* for 4 days (referred to here as the host), whereas the CD45.1^+^ parabiont was left uninfected (Fig. 3c). A group of uninfected parabiotic mice was used as an additional control (Fig. 3c). The ratio of parabiont:host NK cells in the blood was 1, confirming successful chimerism (Fig. 3d). In addition, this ratio was maintained following infection of one parabiont (Fig. 3d). We then assessed the SILP of the parabionts. As a positive control for tissue-resident cells, we examined the CD127^+^NK1.1^−^ ILC population. Consistent with a previous study, CD127^+^ ILCs were almost entirely host-derived, with a parabiont:host ratio of 0.038 (Fig. 3e).^11^ SILP NK cells were also largely tissue-resident in uninfected mice indicated by a parabiont:host ratio of ~ 0.25 (Fig. 3e), confirming previously published data.^11^

We next examined changes to the ILC compartment following *Hpb* infection. At day 4 post infection, the frequency of SILP CD127^+^ ILCs trended downward, whereas the frequency of CD127^−^NK1.1^+^ NK cells significantly increased in infected, but not uninfected, parabionts (Fig. 3f). Consistently, CD127^+^ ILCs remained predominantly tissue-resident with a small, but significant increase in donor-derived cells between but not within parabiotic mice (Fig. 3e). Notably, *Hpb* infection led to an increase in the ratio of parabiont:host NK cells that reached close to 1, indicating that upon infection there was a significant recruitment of NK cells from the circulation to the SILP (Fig. 3e). Furthermore, when we distinguished NK cells based on CD49a and CD49b we found no change in CD49a^+^ trNK cell numbers upon infection, but a significant increase in CD49b^+^ cNK cells in the infected parabionts (Fig. 3g). Overall, these data establish CD49a and CD49b as a valid method to discriminate tissue-resident and circulating NK cells in the SILP. Furthermore, our studies demonstrate that NK cell accumulation during *Hpb* infection results from the recruitment of a circulating CD49b^+^ population.

### A Type 1 immune transcriptional signature dominates the tissue-invasive stage of *Hpb* infection

**Fig. 4:**
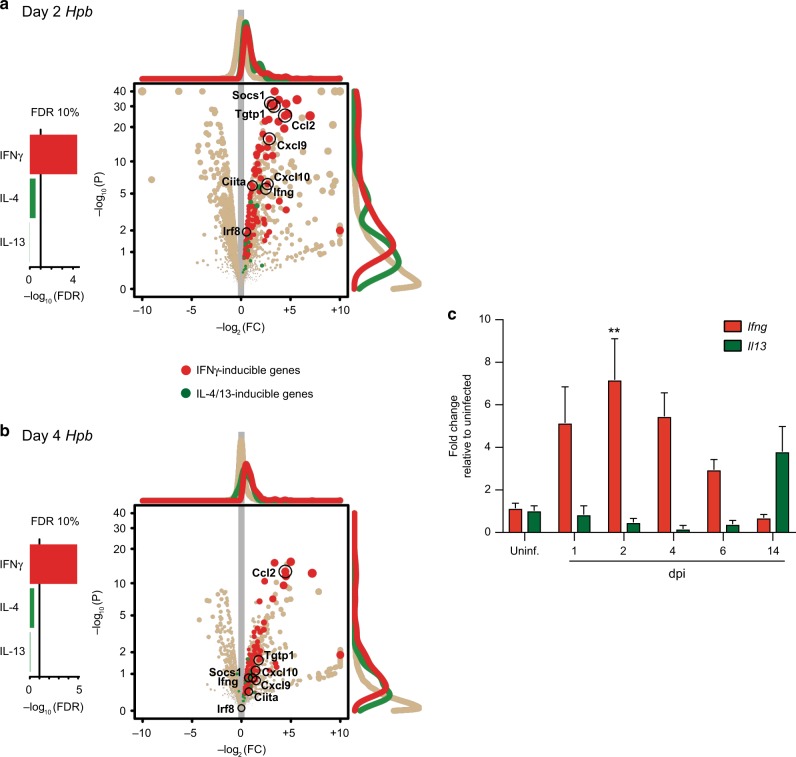
A Type 1 immune response dominates early *Hpb* infection. RNA sequencing results comparing duodenal tissue from uninfected to *Hpb*-infected mice. IFNγ-inducible genes (red) are indicative of a Type 1 immune response, while IL-4/13-inducible genes (green) represent a Type 2 immune response. Pathway analyses show response to cytokine (IFNγ; Gene Ontology’s GO:0034097), response to Interleukin-4 (IL4; GO:0070670), and response to Interleukin-13 (IL-13; GO:0035962) (left panels). Statistical significance chosen at the FDR 10% level. Volcano plot shows *P* value (*P*) of gene expression fold change (FC) for each gene (dot) (right panels). Histograms of data points represented as curves. Selected genes are highlighted. **a** Day 2 *Hpb* infection vs uninfected. **b** Day 4 *Hpb* infection vs uninfected (*n* = 2 mice per group). **c** Fold change relative to uninfected for *Ifng* and *Il13* mRNA expression in uninfected and *Hpb-*infected WT duodenal tissue (*n* = 2–11). **c** Data were analyzed by one-way ANOVA with Dunnet’s post test for multiple comparisons, using uninfected as the control group (**p < 0.01). Error bars, SEM.

Although *Hpb* infection is generally thought to drive an exclusive Type 2/regulatory immune response, the early recruitment of NK cells, a leukocyte generally associated with Type 1 immune responses, prompted us to determine whether this result was representative of a global change in gene expression pattern not previously described during the tissue-invasive stage of *Hpb* infection. Therefore, we performed RNA Sequencing (RNAseq) analysis on whole duodenal tissue comparing uninfected with 2 and 4 dpi samples. Notably, IFNγ-inducible genes such as *Cxcl9*, *Cxcl10*, *Ccl2*, and *Socs1* were significantly increased compared with IL-4/IL-13-induced genes such as *Stat6* and *Gata3* at both 2 and 4 dpi relative to uninfected mice (Fig. 4a, b). The sequencing results were consistent with qRT-PCR analysis showing increased *Ifng* mRNA expression during early infection time points (Fig. 4c). By contrast and as expected, an increase in *Il13* mRNA occurred at 2 weeks post infection, a time point at which adult worms have left the mucosa and have colonized the intestinal lumen (Fig. 4c). These data indicated a dominant Type 1 cytokine *milieu* during *Hpb* infection may be responsible for recruiting circulating NK cells.

### NK cell recruitment to the SILP occurs in an IFNγ-dependent, CXCR3-independent manner during *Hpb* infection

**Fig. 5:**
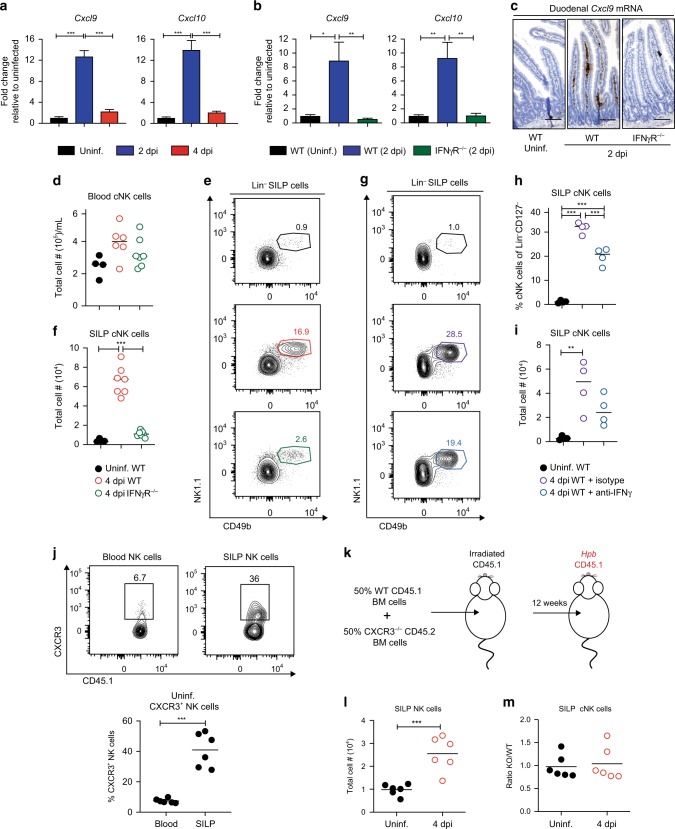
IFNγ-dependent, CXCR3-independent recruitment of NK cells to the SILP during *Hpb* infection. **a** Fold change in *Cxcl9* and *Cxcl10* mRNA expression in WT duodenal tissue of WT mice relative to uninfected WT duodenal tissue at 2 and 4 dpi (*n* = 7–10). **b** Fold change in *Cxcl9* and *Cxcl10* mRNA expression in WT and IFNγR^−/−^ duodenal tissue relative to WT uninfected duodenal tissue at 2 dpi (*n* = 4–7). **c** RNA Scope for *Cxcl9* transcripts, in brown, in the duodenal tissue of uninfected WT and 2 dpi of WT and IFNγR^−/−^ mice (scale bar, 100 μm). **d** Total numbers per mL of blood NK cells (CD127^−^NK1.1^+^CD49b^+^) in uninfected WT or 4 dpi of WT and IFNγR^−/−^ mice. **e** Representative contour plots and **f** total numbers of cNK cells in the SILP of uninfected WT or 4 dpi of WT and IFNγR^−/−^ mice. **g** Representative contour plots, **h** frequency, and **i** total numbers of cNK cells in the SILP of uninfected mice, or 4 dpi of mice treated with anti-IFNγ-blocking antibody or isotype control. **j** Representative contour plots and frequencies of CD127^−^NK1.1^+^CXCR3^+^ NK cells in the blood and SILP of uninfected WT mice. **k** BM chimera experimental design. **l** Total cell number of CD127^−^NK1.1^+^ NK cells in uninfected and 4 dpi BM chimera mice. **m** Ratio of KO/WT SILP cNK cells in uninfected and 4 dpi of BM chimera mice. Data shown are pooled from three **a** and two **b**, **d**–**f**, **j**, **l**, **m** independent experiments and representative of two independent experiments **g**–**i**. **a**, **b**, **d**, **f**, **h**, **i** Data were analyzed by one-way ANOVA with Tukey’s post test for multiple comparisons. **j**, **l**, **m** Data were analyzed using an unpaired parametric *t* test (*p < 0.05, **p < 0.01, ***p < 0.001). Each dot represents an individual mouse. Error bars, SEM.

Following *Hpb* infection, an increase in IFNγ-inducible genes coincided with an accumulation of NK cells. These data led us to test whether IFNγ signaling is required for NK cell recruitment to the SILP during *Hpb* infection. CXCL9 and CXCL10 are both IFNγ-inducible chemokines that bind to the non-redundant CXCR3 receptor and are important for NK cell recruitment to the draining lymph nodes following *orthopoxviru*s infection and lung following influenza A virus infection.^18–20^ Consistent with our RNAseq results, a significant induction in gene expression of these two chemokines occurred at 2 dpi in duodenal tissue samples (Fig. 5a). This result correlated with an increase in CXCL9 protein secretion from whole duodenal tissue at 2 dpi (Fig. S3), which was lost in the absence of IFNγ signaling (Fig. 5b). In order to determine where the CXCL9 was being produced within the duodenum, we visualized *Cxcl9* transcripts using in situ hybridization RNAScope technology. Upon infection, *Cxcl9* transcripts (shown in brown) localized within the lamina propria in an IFNγR-dependent manner, and not along the epithelial border (Fig. 5c), consistent with a non-epithelial source of CXCL9.

In order to assess the importance of IFNγ signaling in NK cell recruitment, we infected IFNγ receptor knock out (IFNγR^−/−^) mice with *Hpb*. We observed no difference in the number of NK cells in the blood between WT and IFNγR^−/−^ mice at 4 dpi (Fig. 5d). In contrast, there was a significant decrease in the frequency and total number of cNK cells at 4 dpi in the SILP of IFNγR^−/−^ mice relative to WT mice (Fig. 5e, f). In order to confirm the requirement for IFNγR in NK cell recruitment during *Hpb* infection was not owing to differences in microbiota between WT and IFNγR^−/−^ mice, we took two different approaches. We first co-housed WT and IFNγR^−/−^ mice for 4 weeks prior to infection. Infection of co-housed WT and IFNγR^−/−^ mice maintained the requirement for IFNγR-signaling in NK cell recruitment (Fig. S4). As a complementary approach, we treated WT mice with an anti-IFNγ-blocking antibody and assessed NK cell recruitment. Similar to our genetic approaches, blockade of IFNγ resulted in a significant decrease in the frequency of cNK cells in the SILP compared with the infected isotype control-treated group, which correlated with total cell numbers (Fig. 5g–i).

To demonstrate a direct role for CXCL9 and CXCL10 on NK cell recruitment, we first analyzed CXCR3 expression on SILP NK cells. When compared with blood NK cells, there was a higher frequency of NK cells in the SILP that expressed CXCR3, suggesting a possible mechanism of NK cell recruitment to the SILP (Fig. 5j). To directly test this possibility, we generated mixed bone marrow chimeric mice in which lethally irradiated CD45.1^+^ WT mice were reconstituted with a 1:1 ratio of bone marrow cells from CD45.1^+^ WT and CD45.2^+^ CXCR3^−/−^ mice (Fig. 5k and Fig. S5). Consistent with our previous experiments, NK cell number increased in the SILP upon infection (Fig. 5l). However, when we compared the ratio of CXCR3^−/−^ cNK cells with WT cNK cells in the SILP, the ratio was close to 1, indicating that both the WT and CXCR3^−/−^ NK cells could equally be recruited to the SILP during *Hpb* infection (Fig. 5m). Overall, these data indicate that NK cells are recruited to the SILP in an IFNγ-dependent, but CXCR3-independent manner during *Hpb* infection.

### Intestinal NK cells limit tissue injury during *Hpb* infection without impacting parasite fitness

**Fig. 6:**
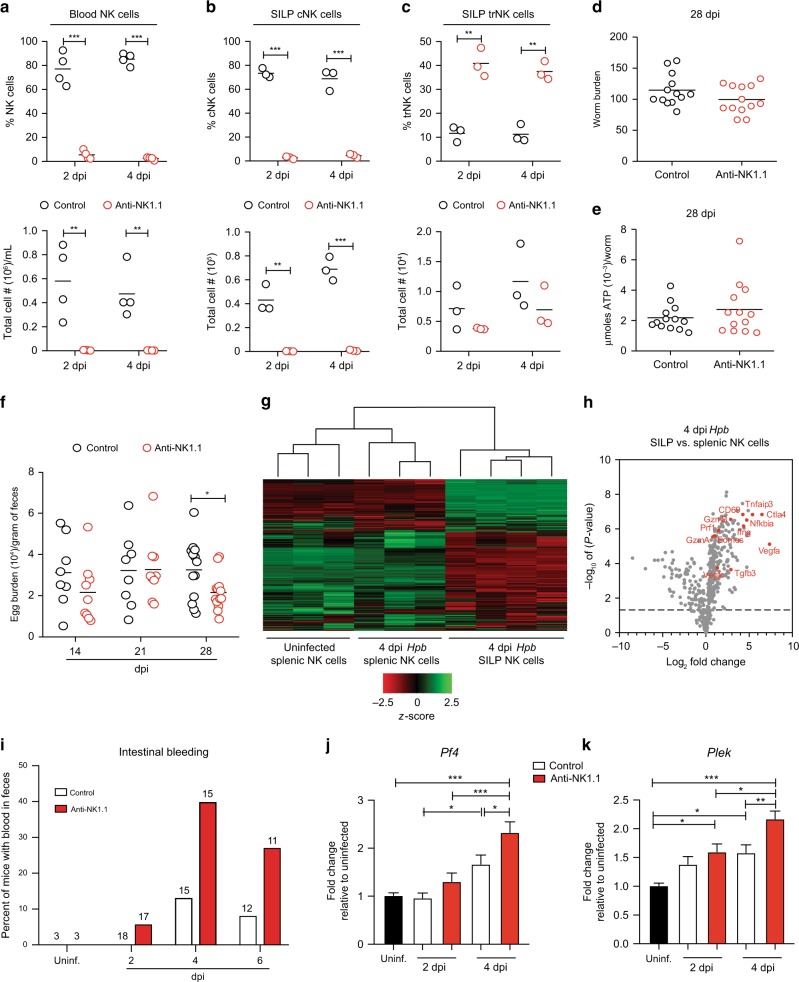
SILP NK cells limit tissue morbidity during *Hpb* infection. **a** Frequency and total cell number of CD127^−^NKp46^+^ NK cells in the blood of WT mice treated with anti-NK1.1 or control antibody at 2 and 4 dpi. **b** Frequency and total cell number of CD127^−^NKp46^+^CD49b^+^ cNK cells, and **c** CD127^−^NKp46^+^CD49a^+^ trNK cells in the SILP of WT mice treated with anti-NK1.1 or control antibody at 2 and 4 dpi. **d** Number of adult worms in the intestinal lumen and **e** μmoles of ATP/worm isolated from WT mice at 28 dpi treated with anti-NK1.1 or control antibody. **f** Egg burden in the feces 14, 21, and 28 dpi of WT mice treated with anti-NK1.1 or control antibody. **g** Heat map and hierarchical clustering of mRNA comparing splenic NK cells from uninfected mice with splenic and SILP NK cells from mice at 4 dpi. **h** Volcano plot comparing gene expression between SILP NK cells and splenic NK cells isolated from *Hpb*-infected mice at 4 dpi. Each dot represents an individual gene; selected genes are highlighted in red. **i** Hemoccult test in feces of uninfected and *Hpb*-infected WT mice treated with anti-NK1.1 or control antibody. Numbers represent mice per group at each time point. **j** Fold change relative to uninfected in *Pf4* and **k** *Plek* mRNA expression in duodenal tissue of uninfected, 2 and 4 dpi WT mice treated with anti-NK1.1 or control antibody (*n* = 9–13). Data shown are pooled from three **d**–**f**, **i** and four **j**, **k** independent experiments. **a**–**f** Data were analyzed using an unpaired parametric t-test comparing each anti-NK1.1 treated group to its respective control-treated group. **g** Hierarchical clustering was performed using Pearson Uncentered Correlation analysis. **h** Genes above the dashed lines are significantly different, *p* < 0.05. **j**, **k** Data were analyzed by one-way ANOVA with Tukey’s post test for multiple comparisons (*p < 0.05, **p < 0.01, ***p < 0.001). Each dot represents an individual mouse. Error bars, SEM.

Given the localization of NK cells in proximity to the larvae-embedded granulomas of infected mice, we hypothesized that NK cells contribute to host defense against *Hpb* infection. To test this possibility, we depleted NK cells by anti-NK1.1 antibody treatment. Administration of anti-NK1.1 resulted in almost complete depletion of blood NK cells compared with isotype control-treated animals (Fig. 6a). Interestingly, anti-NK1.1 treatment selectively depleted CD49b^+^ cNK cells and not CD49a^+^ trNK cells from the intestine of *Hpb*-infected mice (Fig. 6b, c). We took advantage of this selective depletion to test the role of cNK cells in the anti-helminth immune response. Surprisingly, cNK cell depletion had no impact on worm burden (the number of adult worms in the lumen of the small intestine) at 28 dpi (Fig. 6d). We next assessed worm fitness and fecundity in the presence or absence of cNK cells. The amount of parasite-derived ATP can be used as a measure of worm fitness.^21,22^ However, no difference in ATP production from adult *Hpb* extracted from control or cNK cell-depleted mice was observed (Fig. 6e). Adult *Hpb* produce and disseminate eggs in order to continue their life cycle. As a second test of parasite fitness, we assessed worm fecundity by counting the number of eggs per gram of feces from infected mice. With the exception of a small, but significant decrease in egg burden at 28 dpi, no differences in egg burden were detected between control and NK cell-depleted mice (Fig. 6f). Overall, these data suggest that cNK cells do not significantly contribute to host resistance to *Hpb* infection.

In addition to pathogen resistance, the host can execute defense mechanisms that limit tissue damage and promote organism fitness without a direct impact on parasite burden, a phenomenon known as disease tolerance.^23^ As our data suggested that SILP NK cells were not directly impacting *Hpb* function or survival, we speculated that NK cells may be contributing to a tissue protective response. Thus, we used NanoString technology in order to obtain a targeted gene expression signature by NK cells at the site of infection. We purified NK cells from the SILP of *Hpb*-infected mice and compared their gene expression with splenic NK cells isolated from uninfected and infected mice. Regardless of infection status, splenic NK cells clustered together, whereas SILP NK cells clustered separately (Fig. 6g). Overall, these data indicate that the gene expression profile induced in NK cells by *Hpb* infection is specific to cells at the site of infection. Consistent with our phenotyping results, SILP NK cells expressed increased *Eomes* and *Ifng* mRNA as well as genes coding for cytotoxic effector molecules such as *GzmA*, *GzmB,* and *Prf1* compared with splenic NK cells (Fig. 6h). However, other top genes highly expressed in SILP NK cells included immunoregulatory and inhibitory co-receptor genes such as *Ctla4*, *Tigit*, *Cd274*, *Tnfaip3,* and *Nfkbia,* suggesting an immunoregulatory phenotype. Notably, genes previously demonstrated to regulate vascular remodeling and vascular integrity such as *Vegfa*, *Vegfc,* and *Tgfb3* were also enriched in intestinal NK cells^24,25^ (Fig. 6h and Table S1).

As our gene expression results were reminiscent of previous studies showing that uterine NK cells enriched for *Ifng* and *Vegfa* expression promote vascular integrity during pregnancy,^24,26^ we tested whether NK cells infiltrating the intestine during *Hpb* infection may be playing a similar role. To this end, we assessed intestinal bleeding—as determined by heme detection in fecal pellets—following *Hpb* infection of cNK cell-depleted mice and compared them with the isotype control-treated mice. Notably, an increase in the frequency of mice with intestinal bleeding was present upon infection of cNK cell-depleted mice compared with controls (Fig. 6i). Consistent with increased vascular stress and/or injury, duodenal tissue from cNK cell-depleted mice infected with *Hpb* also expressed higher levels of transcripts associated with platelet-specific activation genes including platelet factor 4 (*Pf4*) and pleckstrin (*Plek*) at time points when intestinal bleeding was most prevalent (Fig. 6j, k). Collectively, these data suggest that NK cells contribute to maintaining intestinal tissue integrity and host tolerance during the early tissue-invasive stages of *Hpb* infection.

## Discussion

Many soil-transmitted helminths such as *Trichinella, Ascaris, and Trichuris spp*. first encounter and penetrate host tissue at the intestinal barrier. Although tissue invasion causes significant inflammation and structural damage, the consequences of infection are often insidious and fail to evoke discrete sickness behavior. This remarkable disease tolerance to infection suggests that the mammalian host defense system has evolved robust mechanisms to limit tissue damage and endure these multicellular parasite challenges. Understanding the pathways involved in disease tolerance to intestinal helminth infection may not only provide strategies to mitigate the morbidities directly associated with infection, but may also inform strategies to improve mucosal healing across diverse settings of gut inflammation and tissue injury.

To gain more insight into host tolerance to helminth infection, we examined the early stages of *Hpb* infection, a natural parasitic roundworm of mice.^27^ Following entry into the small intestine, *Hpb* larvae migrate through the intestinal epithelium where they embed and mature into adult worms within the submucosa. After maturation, *Hpb* emigrates from the intestinal parenchyma and colonizes the intestinal lumen where it reproduces to continue its life cycle. We and others have previously described a robust Type 2-dominant immune response including recruitment of IL-4-producing Th2 cells, eosinophils, and activation of ILC2s and alternatively activated macrophages detectable as early as day 5 post-*Hpb* infection.^28–30^ However, few studies have examined earlier time points of *Hpb* infection. Unexpectedly, we found that prior to the onset of a detectable Th2 response, acute *Hpb* infection elicited a robust accumulation of mature, activated NK cells. NK cell accumulation was unique to the ILC compartment as no increase in ILC1, ILC2, or ILC3 subsets occurred at this stage of infection. Through the use of immunophenotyping and parabiosis experiments we determined that the increase in intestinal NK cells was due to the recruitment of circulating CD49a^−^CD49b^+^Eomes^+^ cells. Our results build on a previous study by Sojka et al., demonstrating in various non-intestinal organs that the integrin subunits CD49b and CD49a delineate circulating and tissue-resident murine NK cells, respectively.^17^ The specificity of the response, their increased activation state and their proximity to the granuloma suggests that cNK cells may be directly responsive to *Hpb* itself or its excretory-secretory (ES) products. Consistent with this possibility, human NK cells have been previously shown to produce IFNγ in response to ES products secreted by the hookworm *Necator americanus*.^31,32^

The unexpected accumulation of cNK cells in response to helminth infection prompted us to examine global gene expression changes during the early stages of *Hpb* infection and to identify potential mechanisms of NK cell recruitment. RNAseq of whole duodenal tissue preparations during early *Hpb* infection revealed an enrichment of IFNγ-inducible transcripts compared with quintessential IL-4/13 genes known to be induced during the chronic stages of *Hpb* infection. Consistent with these results, Nusse et al.^10^ recently described an early induction of IFNγ signaling surrounding the *Hpb* granuloma that drove a fetal-like Ly-6a^+^ intestinal stem cell reversion to promote epithelial regeneration. Our RNA sequencing studies also indicated a robust induction of *Ly6a* transcripts during *Hpb* tissue invasion that temporally correlated with peak IFNγ transcription and downstream target gene expression. Based on this collective work, it will be important to investigate the signal that stimulates IFNγ production and the precise location of its induction. Likely stimuli are microbe-associated molecular patterns (MAMPs) derived from the commensal microbiota, well-known to drive a Type 1 immune response. *Hpb*-induced barrier breakdown may increase the exposure of MAMPs to luminal-probing CX_3_CR1^+^ mononuclear phagocytes as well as intestinal epithelial cells (IECs) and their subsequent production of IL-1β, IL-12, and/or IL-18 may be important for driving IFNγ induction by tissue-resident innate-like intraepithelial T lymphocytes, ILC1s or recruited NK cells.^33^ Alternatively, stress-induced ligands upregulated by IECs during infection may engage activating receptors on NK cells to drive activation.^33^ It is interesting to consider that *Hpb*-mediated induction of a Type 1 immune response may not only limit collateral damage associated with microbiota exposure during parasite tissue invasion, but may also be exploited by the parasite to limit the magnitude of a Type 2 response that could prevent larval maturation and/or accelerate adult worm expulsion by the “weep and sweep” response, the latter of which is established to occur during *T. muris* infection.^4^

The increase in IFNγ-inducible chemokines such as CXCL9 and CXCL10 during *Hpb* infection led us to anticipate a critical role for CXCR3 in NK cell recruitment to the intestine.^18,20,34^ Indeed, we found CXCR3 expression to be enriched on intestinal compared with blood NK cells and this chemokine receptor has been shown to be important for NK cell recruitment to the inflamed lymph nodes in the context of *orthopoxvirus* infection.^18^ However, using a mixed bone marrow chimera approach, we demonstrated that NK cell expression of CXCR3 was dispensable for NK cell recruitment to the SILP during *Hpb* infection. Alternatively, it has been recently suggested that CXCR3 may be more important for the recruitment of immature NK cells, whereas CX_3_CR1 is more important for mature NK cell recruitment as their expression levels correlate with early and later stages of NK cell differentiation, respectively.^14,35–37^ Given the mature phenotype of the NK cells present in the intestine during *Hpb* infection, we speculate that CX_3_CR1 could be a mechanism by which NK cells are recruited. Our RNAseq data also highlighted increases in other NK cell chemokines such as CCL2 and CCL4. Indeed, these chemokines have been shown to recruit NK cells in the context of diverse parasitic and fungal infections.^38,39^ Considering that the mechanisms of NK cell recruitment occurs in an organ and pathogen-specific manner, future studies will need to be performed to determine, which chemotactic signal(s) drive helminth-induced NK cell recruitment.

Given the proximity of NK cells to *Hpb* larvae in situ, we hypothesized that they would limit parasite burden and/or fitness. However, upon effective depletion of cNK cells, we observed no significant difference in adult worm burden or parasite fitness compared with NK cell replete animals. As *Hpb* infection is very well tolerated by the host, few studies have examined the mechanisms that limit tissue damage during infection with this pathosymbiont. We therefore asked whether cNK cells played a role in host tolerance rather than host resistance.^3^ Intestinal bleeding is the most common co-morbidity associated with human helminth infection.^1^ As transcriptional profiling of purified intestinal NK cells showed an enrichment for genes that support vascular integrity and endothelial cell survival such as *Ifng*, *Vegfa*, *Vegfc,* and *Tgfb3*, we assessed whether these cells also contribute to vascular health during *Hpb* infection. Indeed, depletion of cNK cells increased the incidence of intestinal bleeding within the first week of *Hpb* infection relative to NK cell replete mice. Corroborating these data, an increase in *Plek* (pleckstrin) and *Pf4* (platelet factor 4) transcripts occurred in the absence of cNK cells. Pleckstrin is a known marker of platelet activation (among other cells) and has been suggested to play a role in proper platelet aggregation.^40^ Platelet factor 4 has been shown to inhibit endothelial proliferation and to have both anti- and pro-coagulation properties.^41,42^ As uterine NK cells are well-established to play a critical role in maintaining vascular integrity and producing angiogenic factors during murine and human pregnancy,^24,26^ our data point towards a similar role for NK cells in the context of an infection where limiting the damage incurred by invasion of a multicellular parasite may usurp the more conventional cytotoxic functions required to eliminate intracellular pathogens. However, our studies do not rule out the possibility that NK cells recruited to the intestine during *Hpb* infection also exert cytotoxic activity to rapidly eliminate stressed cells that allows for rapid replacement or repair of the damaged endothelium.^43^ As our studies took advantage of targeting cNK cells using antibody-mediated depletion, it will be important to determine whether this subset is functionally distinct from its tissue-resident counterpart (i.e., trNK cells) or whether complete loss of the NK cell lineage would lead to a greater compromise in host tolerance to *Hpb* infection. Overall, we have demonstrated that the immune response to *Hpb* is more diverse than previously appreciated and that IFNγ-dependent NK cell recruitment contributes to disease tolerance in the intestine. In addition, our work also provides the impetus for further investigation into the mechanisms that maximize tissue integrity and host fitness in the context of enteric helminth infection.

## Materials and methods

### Mice

All experiments were performed in accordance with the McGill University Animal Care Committee. CD45.1, CD45.2, 4get/KN2, RORγt ^GFP/+^, RORγt^GFP/GFP^, IFNγR^−/−^, and CXCR3^−/−^ mice (all on the C57BL/6 background), and BALB/c mice were bred and maintained under specific pathogen-free conditions. Female and male mice of 6–16 weeks of age were used.

### *Hpb* infection and antibody treatment

Mice were infected by gavage with 200 L3 stage *Hpb* larvae diluted in sterile water, killed at the indicated time points, and tissues were harvested for analysis. For IFNγ-blocking experiments, mice were treated with 500 μg of anti-IFNγ (clone XMG1.2; BioXcell) or isotype (clone HRPN; BioXcell) every day, starting 2 days prior to infection. For NK cell depletion experiments, mice were treated with 200 μg of anti-NK1.1 depletion antibody (clone PK136, BioXcell) or isotype (clone C1.18.4; BioXcell) on the day of infection and every second day until day 12 dpi.

### Co-housing

WT and IFNγR^−/−^ mice were co-housed for 4 weeks prior to and during infection. Mice were harvested 4 dpi with *Hpb*.

### SILP cell extraction

The first 10 cm of the duodenum was used to extract lamina propria cells. Fat tissue and Peyer’s patches were removed, the tissue was cut longitudinally and washed in cold Hank’s Balanced Salt Solution (HBSS) + EDTA buffer (HBSS) supplemented with 5 mM EDTA, 10% heat-inactivated fetal bovine serum (FBS) and 15 mM HEPES). The tissue was cut in 0.5–1 cm pieces, placed in 15 mL of pre-warmed HBSS + EDTA buffer and incubated at 37 °C, with shaking at 250 rpm, for 20 min. The tissue was filtered through a 100 μm filter in order to remove the intraepithelial lymphocytes and epithelial cells and this process was repeated a second time. The tissue was then washed twice with 15 mL of cold HBSS buffer (HBSS supplemented with 2% FBS and 15 mM HEPES), followed by centrifugation for 2 min at 1800 rpm at 4 °C. Following the second wash, buffer was decanted, and excess liquid was removed. The tissue was digested in 5 mL of digestion buffer (RPMI 1640 supplemented with 10% FBS, 15 mM HEPES, 100 U/mL of DNAse and 200 U/mL of Collagenase VIII) for 25 min at 37 °C, with shaking at 250 rpm. The digestion was stopped by adding 35 mL of cold R10 buffer (RPMI 1640 supplemented with 10% FBS, 15 mM HEPES, 1% l-glutamine and 1% penicillin/streptomycin). The tissue was crushed, passed through a 100 μm filter and centrifuged at 2500 rpm for 7 min at 4 °C. The cells were then resuspended in R10 buffer and enumerated using Trypan Blue.

### Liver extraction protocol

Mice were bled and perfused with 20 mL of PBS. The liver was removed, cut into small pieces and digested in 10 mL of digestion buffer (RPMI 1640 supplemented with 10% FBS, 15 mM HEPES, 150 U/mL of DNAse and 500 U/mL of Collagenase IV) for 35 min at 37 °C, with shaking at 250 rpm. Every 10 minute during the digestion, the samples were shaken vigorously by hand. The digestion was stopped by adding 35 mL of cold R10 buffer. The samples were filtered twice using a 100 μm filter and centrifuged at 300 *g* for 5 min at 4 °C. The supernatant was aspirated, 30 mL of R10 buffer was added and samples were centrifuged at 300 *g* for 5 min at 4 °C. The cells were then lysed for 3 min in 1× red blood cell lysing buffer, centrifuged at 300 *g* for 5 min at 4 °C, resuspended in R10 buffer and enumerated using Trypan Blue.

### Flow cytometry

For cells from the SILP and liver, cells were extracted as described above. For the blood, the spleen and the BM, the cells were lysed in 1× red blood cell lysing buffer prior to counting and staining. For ex vivo restimulation, cells were incubated with 50 ng/mL phorbol myristate acetate and 1 μg/mL ionomycin for 4 h at 37 °C with 5% CO_2_ in the presence of BD GolgiStop. Cell suspensions were incubated with a fixable Viability dye (eFluor 506,eBioscience) for 25 min at 4 °C. Cells were then incubated with Fc block (7 min at 4 °C), followed by staining (for 30 min at 4 °C) with the following antibodies in appropriate combinations of fluorophores. From Invitrogen: B220 (RA3–6B2), CD3 (145–2C11), CD45.1 (A20), CD45.2 (104), CD127 (A7R34), Eomesodermin (Dan11mag), CXCR3 (CXCR3-173), GATA-3 (TWAJ), IFNγ (XMG1.2), NK1.1 (PK136), NKp46 (29A1.4) and Tbet (eBio4b10). From Biolegend: CD11b (M1/70), CD49a (Ha31/8), CD49b (HMa2), Granzyme B (GB11), and RORγt (Q31-378). Cells were fixed with 2% paraformaldehyde for 12 min at 4 °C prior to analysis. For staining for intracellular proteins, cells were fixed and permeabilized with the FoxP3 Fix/Perm kit (eBiosciences) according to the manufacturer’s instructions. Data were acquired with a FACS Canto II or LSR Fortessa (BD Biosciences) and analyzed using FlowJo software (TreeStar).

### RNA extraction and qRT-PCR

Whole duodenal tissue was harvested and flash frozen and stored at −80 °C. Total RNA was extracted by crushing the tissue using a pestle and mortar on dry ice and extracted by using the QIAGEN RNeasy Mini Kit as per the manufacturer’s instructions. In all, 1 μg of RNA was reverse-transcribed by QuantiTect Reverse Transcription Kit (Thermofisher). Relative gene expression was measured by qRT-PCR for *Ifng, Il13*, *Cxcl9, Cxcl10, Plek,* and *Pf4*. The expression of the gene of interest for each cDNA sample was normalized to the gene expression of the housekeeping gene *Hprt* and expressed as a fold change relative to uninfected samples. Samples were excluded when the *Hprt* Ct value was greater than one cycle different from the average *Hprt* Ct value for the group. The following primers were ordered from Integrated DNA Technologies. CXCL9 forward primer (cttttcctcttgggcatcat) and reverse primer (gcatcgtgcattccttatca). CXCL10 forward primer (gcaccatgaacccaagtg) and reverse primer (ttcatcgtggcaatgatctcaaca). HPRT forward primer (aggacctctcgaagtgttgg) and reverse primer (aacttgcgctcatcttaggc). IFNγ forward primer (ttcttcagcaacagcaaggc) and reverse primer (actccttttccgcttcctga). IL-13 forward primer (attgcatggcctctgtaacc) and reverse primer (tgagtccacagctgagatgc). Platelet factor 4 (Pf4) forward primer (tgtgaagaccatctcctctgg) and reverse primer (ggcagctgatacctaactctcc). Pleckstrin (Plek) forward primer (tcacttgagaggctgtgtgg) and reverse primer (ctcagtgattctcggtgtcc).

### Hematoxylin and eosin staining

Ten percent formalin fixed paraffin-embedded duodenal tissue of uninfected and *Hpb*-infected mice were stained with H&E by the Goodman Cancer Research Centre Histology Facility.

### NK cell purification and CFSE labeling

NK cells from the spleen of a CD45.1^+^ mouse were isolated using the EasySep Mouse NK Isolation Kit (Stem Cell, 19855) per the manufacturer’s instructions. Purified NK cells were resuspended at a concentration of 10 × 10^6^ cells/mL and were labeled with CFSE for 5 min in PBS with 5% FBS at room temperature at a final concentration of 5.5 μM. The cells were then washed twice at 4 °C for 7 min and 0.5 million cells were intravenously transferred into CD45.2^+^ recipient mice.

### Confocal microscopy

Swiss rolls were made from 1.5 cm of duodenal tissue of CFSE^+^ NK cells transferred mice. The swiss rolls were frozen in optimal cutting temperature compound and stored at −20 °C. The tissue was sectioned into 4–6 μm slices. The sections were fixed in ice-cold acetone (25% ethanol, 75% acetone) for 5 min and transferred to PBS. The slides were placed in a dark humid chamber at room temperature for staining. The samples were blocked for 1 h in 2% BSA with Fc block (1:100). The blocking solution was aspirated and the slides were stained with CD45.1 (A20) and CD45.2 (104) in 2% BSA for 1 h. Sections were washed in PBS twice for 7 min, stained with the nuclear stain, DRAQ5, for 5 min and mounted using Prolong Gold. Images were taken using a Zeiss LSM780 laser scanning confocal microscope and analyzed using Fiji software.

### RNA Sequencing and data analysis

Extracted RNA was controlled for integrity using the Agilent Bioanalyzer (RNA Analysis), Stranded libraries were prepared using the NEB mRNA stranded library prep Kit (New England Biolabs) and sequenced using HiSeq2500 sequencers (Illumina). Raw reads are clipped for adapter sequence, trimmed for minimum quality (Q30) in 3’ and filtered for minimum length of 32 bp using Trimmomatic.^44^ Surviving read pairs were aligned to Mus_musculus assembly GRCm38 by the ultrafast universal RNAseq aligner STAR^45^ using the recommended two passes approach. Aligned RNA Seq reads were assembled into transcripts and their relative abundance was estimated using Cufflinks and Cuffdiff.^46^ Exploratory analysis was conducted using various functions and packages from R and the Bioconductor project. Differential expression was conducted using both edgeR and DEseq. Terms from the Gene Ontology were tested for enrichment with the GOseq^47^ R package.

### Parabiosis experiments

Parabiosis experiments were performed as previously described.^48^ In brief, CD45.1 and CD45.2 female mice were surgically attached for 4 weeks and chimerism was assessed by blood sample analysis using flow cytometry. In one group, the CD45.2 parabiont was infected with *Hpb,* whereas the CD45.1 parabiont was left uninfected. Uninfected pairs, where both parabionts were uninfected, were used as controls.

### ELISA

The top 5 cm of the duodenum was harvested and fat and Peyer’s patches were removed. The tissue was then cut longitudinally, washed in PBS and excess liquid was removed with absorbent Kimwipes. The tissue was then weighed, placed in 600 μL of PBS and kept on ice. Tissue was homogenized and centrifuged for 5 min at 1800 rpm for 4 °C. The supernatant was collected and transferred into an 1.5 mL tube and centrifuged for 5 min at 13,000 rpm for 4 °C. The supernatant was collected, stored at −20 °C and used for ELISA. The mouse CXCL9/MIG DuoSet ELISA development system from R&D Systems was used per manufacturer’s instructions.

### In situ gene expression analysis

Ten percent formalin fixed paraffin-embedded duodenal tissue were stained using the RNAscope HD brown detection kit as per manufacturer instructions. Slides were imaged at × 10 magnification.

### Bone marrow chimera generation

In all, 11–14-week old WT CD45.1 mice were lethally irradiated with a final dose of 1000 rad (split dose of 500 rad). The same day, mice were i.v. injected with a mixture of 2.5 × 10^6^ BM cells from a CD45.1 WT mouse and 2.5 × 10^6^ BM cells from a CD45.2 CXCR3^−/−^ mouse. The mice were given 12 weeks to reach chimerism prior to *Hpb* infection.

### Parasite fecundity and fitness testing

Mice were infected with *Hpb* and the small intestine was harvested 28 dpi. The adult worms were extracted from the duodenum, counted and placed in 500 μL of cold RPMI media. The worms were homogenized and centrifuged for 7 min at 3500 rpm. The supernatant was collected and the CellTiter-Glo 2.0 assay (Promega) was used to record ATP production per worm by the GloMax Navigator Microplate Luminometer (Promega). ATP (Sigma) was used as a standard. To measure parasite fecundity, feces were collected from infected mice at various time points post infection, weighed and placed in 1 mL saturated NaCl solution. The samples were vortexed and left at room temperature for 24 h and then placed at 4 °C. The eggs were enumerated and normalized to the volume of the supernatant and weight of feces.

### NK cell purification for NanoString analysis

NK cells were isolated from the spleens of uninfected or spleens and small intestines of 4 day *Hpb*-infected WT mice using the EasySep Mouse NK Isolation Kit (Stem Cell technologies) per the manufacturer’s instructions. Total RNA was extracted using the QIAGEN RNeasy Micro-Plus Kit as per the manufacturer’s instructions. SILP NK cells were further purified using FACSAria technology gating on live CD45.2^+^CD3^−^B220^−^CD11b^int^CD127^−^NK1.1^+^ NKp46^+^ cells. Purified NK cells from each organ were lysed and processed per NanoString instructions. Gene expression was quantified using the mouse PanCancer Immune Profiling panel and analyzed using nCounter software.

### Hemoccult test

Feces were collected from uninfected and infected mice at various time points post infection and Hemoccult SENSA kit (Beckman Coulter) was used to assess intestinal bleeding per manufacturer’s instructions.

### Statistical analysis

Data were analyzed using GraphPad Prism software. Specific tests for determining statistical significance are indicated in the figure legends. *P* values < 0.05 were considered statistically significant.

## Supplementary information

Supplementary Information
