# Supplementary material for: NK cell recruitment limits tissue damage during an enteric helminth infection
Source: Mucosal Immunol. 2019 Nov 27;13(2):357–70. doi: 10.1038/s41385-019-0231-8 (PMC7039810; doi:10.1038/s41385-019-0231-8)
Supplement: Supplementary file 1 — Supplementary Information [file 41385_2019_231_MOESM1_ESM.pdf]

Supplementary Figure 1

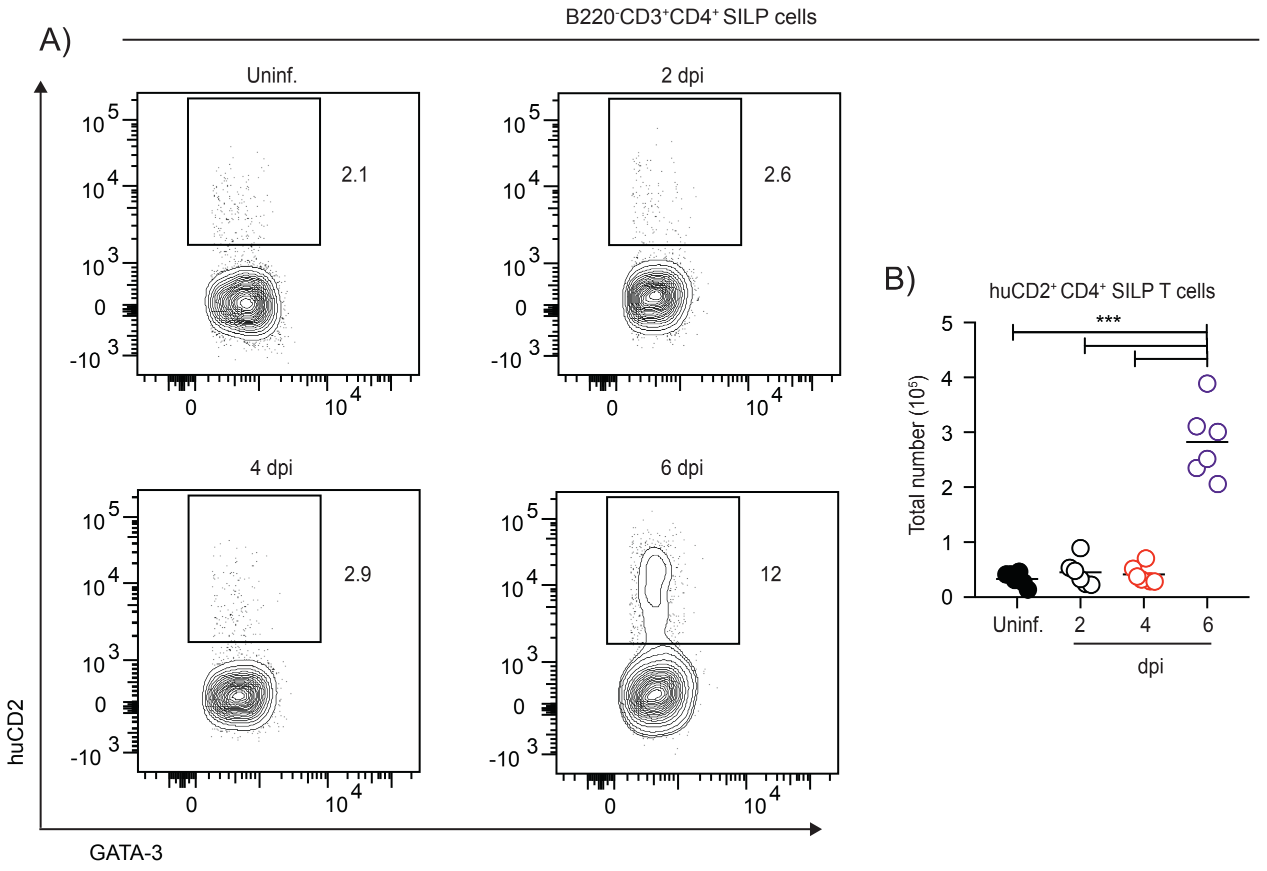

IL-4 producing GATA-3^{+} Th2 cells are first detectable at day 6 in the SILP during *Hpb* infection. **A)** Representative contour plots and **B)** total number of CD45^{+}B220^{-}CD3^{+}CD4^{+}GATA-3^{+}huCD2^{+} T cells in the SILP of uninfected and *Hpb*-infected 4get/KN2 mice at the indicated time points. huCD2 is the reporter for IL-4^{+} secreting cells. **B)** Data pooled from two independent experiments and analysed by one-way ANOVA with Dunnet's post-test for multiple comparisons, using uninfected as the control group (***p < 0.001). Each dot represents an individual mouse.

Supplementary Figure 2

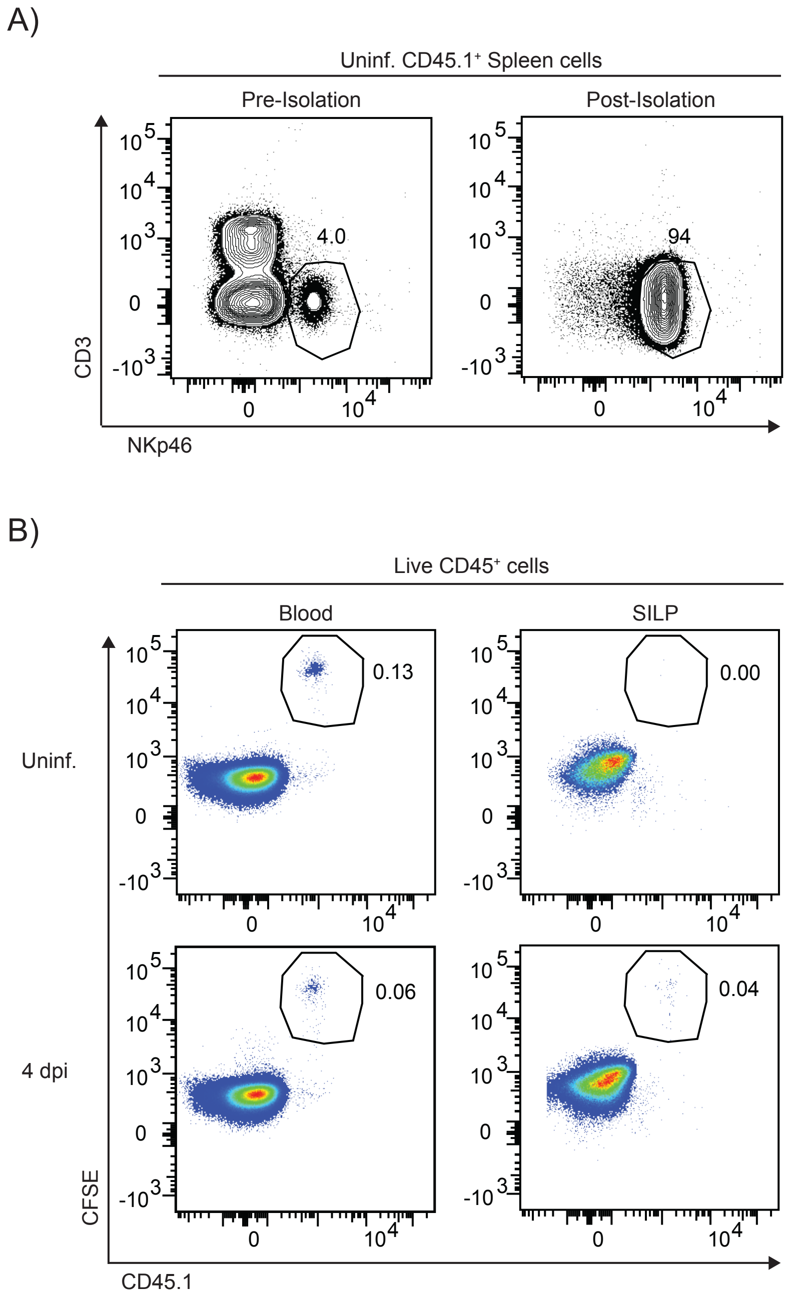

Purity and detection of CFSE⁺ NK cells. Purified CD45.1⁺ splenic NK cells were transferred into CD45.2⁺ recipient mice. **A)** Contour plots of (CD3⁻NKp46⁺) NK cell frequency pre-and post-isolation. **B)** Representative pseudocolour plots of CFSE-labelled CD45.1⁺ cells in the blood and SILP of uninfected and 4 days *Hpb*-infected CD45.2⁺ mice.

Supplementary Figure 3

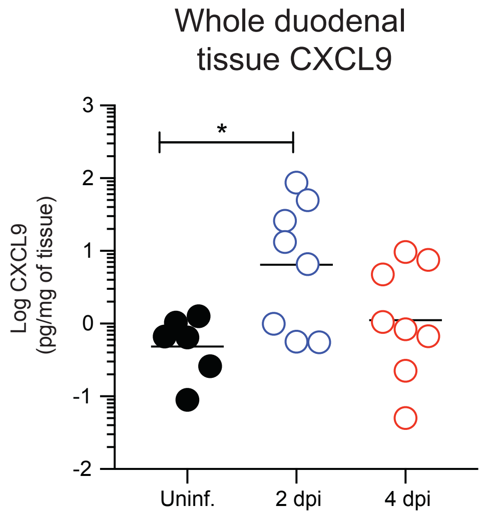

CXCL9 protein is increased in the small intestine during *Hpb* infection. Total CXCL9 protein measured from whole duodenal tissue homogenates from uninfected mice, or 2 and 4 day *Hpb*-infected mice. Data pooled from two independent experiments and analysed by one-way ANOVA with Dunnet's post-test for multiple comparisons, using uninfected as the control group (*$p < 0.05$). Each dot represents an individual mouse.

Supplementary Figure 4

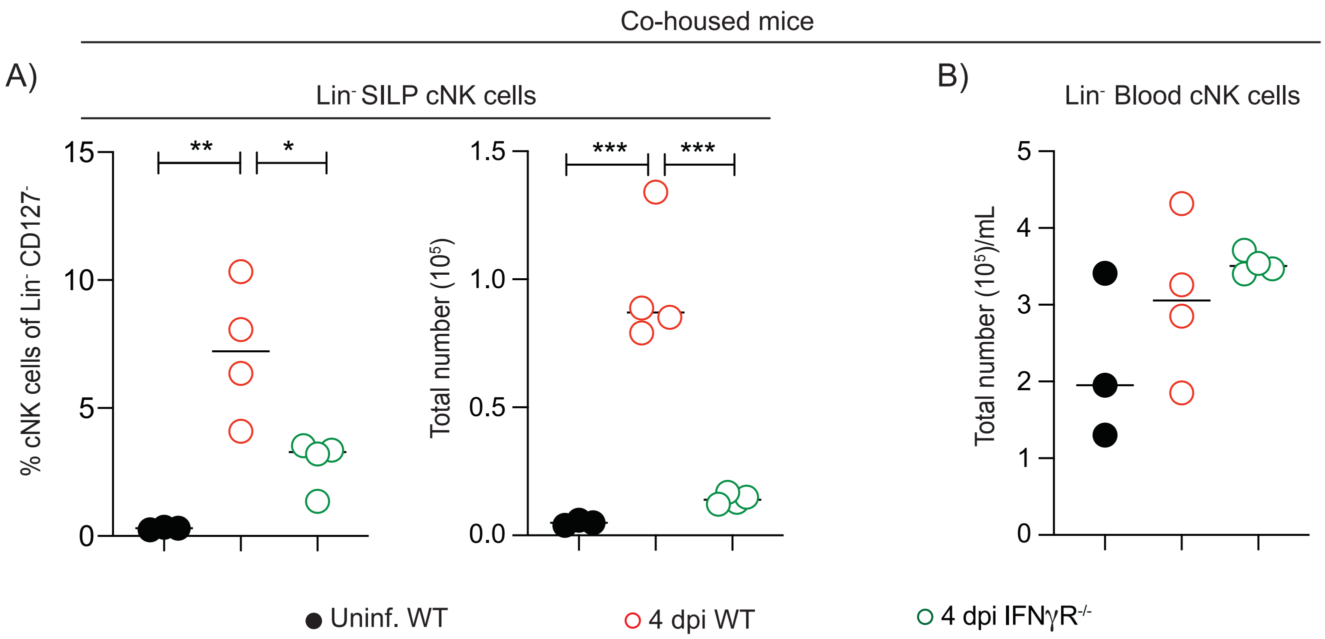

Co-housing does not impact the requirement for IFNγ signaling in cNK cell recruitment to the SILP during *Hpb* infection. A) Frequencies and total numbers of cNK cells in the SILP of uninfected WT, 4 dpi WT and IFNγ$R^{-/-}$ mice after a 4 week co-housing period. **B)** Total numbers of cNK cells per mL of blood in uninfected WT, 4 dpi WT and IFNγ$R^{-/-}$ co-housed mice. Data was analysed by one-way ANOVA with Tukey's post-test for multiple comparisons (*$p < 0.05$, **$p < 0.001$,***$p < 0.001$). Each dot represents an individual mouse.

Supplementary Figure 5

A)

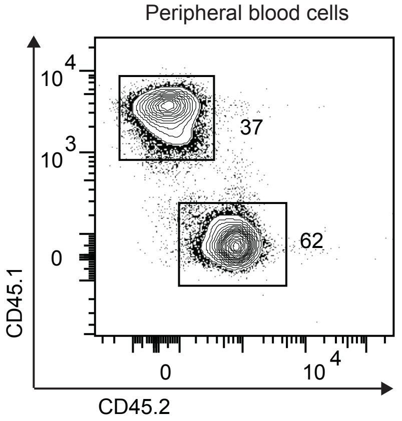

B)

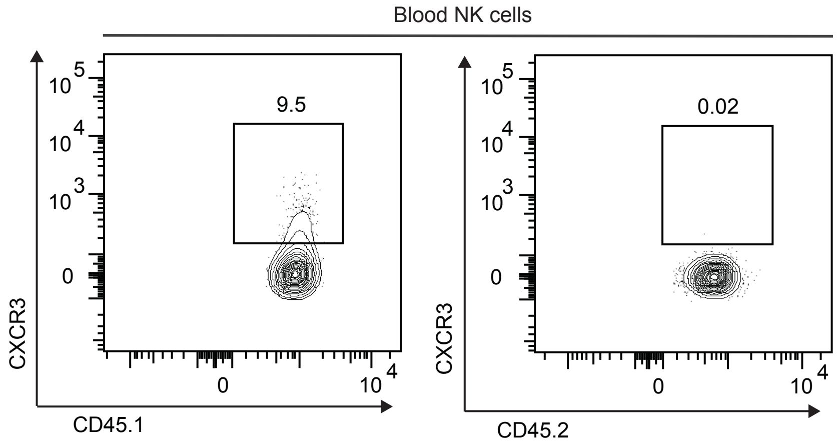

Quality controls for the CXCR3 mixed bone marrow chimera experiments. A) Representative contour plots of the frequency of WT (CD45.1$^+$) and CXCR3$^{-/-}$ (CD45.2$^+$) cells in the blood prior to infection of the chimeric mice 12 weeks after BM transplantation. **B)** Representative contour plots of CXCR3 expression by CD127$^-$NK1.1$^+$ NK cells in the blood of uninfected chimeric mice.
